# Relating Cognition to both Brain Structure and Function: A Systematic Review of Methods

**DOI:** 10.1089/brain.2022.0036

**Published:** 2023-04-04

**Authors:** Marta Czime Litwińczuk, Nelson Trujillo-Barreto, Nils Muhlert, Lauren Cloutman, Anna Woollams

**Affiliations:** Division of Neuroscience and Experimental Psychology, Faculty of Biology, Medicine and Health, University of Manchester, Manchester, United Kingdom.

**Keywords:** cognition, function, multivariate, neuroimaging, review, structure

## Abstract

**Introduction:**

Cognitive neuroscience explores the mechanisms of cognition by studying its structural and functional brain correlates. Many studies have combined structural and functional neuroimaging techniques to uncover the complex relationship between them. In this study, we report the first systematic review that assesses how information from structural and functional neuroimaging methods can be integrated to investigate the brain substrates of cognition.

**Procedure:**

Web of Science and Scopus databases were searched for studies of healthy young adult populations that collected cognitive data and structural and functional neuroimaging data.

**Results:**

Five percent of screened studies met all inclusion criteria. Next, 50% of included studies related cognitive performance to brain structure and function without quantitative analysis of the relationship. Finally, 31% of studies formally integrated structural and functional brain data. Overall, many studies consider either structural or functional neural correlates of cognition, and of those that consider both, they have rarely been integrated. We identified four emergent approaches to the characterization of the relationship between brain structure, function, and cognition; comparative, predictive, fusion, and complementary.

**Discussion:**

We discuss the insights provided in each approach about the relationship between brain structure and function and how it impacts cognitive performance. In addition, we discuss how authors can select approaches to suit their research questions.

**Impact statement:**

The relationship between structural and functional brain networks and their relationship to cognition is a matter of current investigations. This work surveys how researchers have studied the relationship between brain structure and function and its impact on cognitive function in healthy adult populations. We review four emergent approaches of quantitative analysis of this multivariate problem; comparative, predictive, fusion, and complementary. We explain the characteristics of each approach, discuss the insights provided in each approach, and how authors can combine approaches to suit their research questions.

## Introduction

Cognitive function and adaptive behavior rely on structure and dynamics of large-scale neural networks (Friston, 2002). Early cognitive neuroscience separately assessed how properties and characteristics of brain structure and function might impact upon performance of cognitive tasks. Research using the structural modality focused on studying physical properties of the brain, such as cytoarchitecture and neuronal integrity, whereas research using functional approaches assessed characteristics of neuronal activity observed during performance of cognitive tasks and during rest (Rykhlevskaia et al, 2008).

However, in recent years some attempts have been made to integrate the two approaches. Authors have begun to investigate how structure and function of the human brain relate to each other by assessing correspondence between findings from the two modalities (Johansen-Berg et al, 2004; Rykhlevskaia et al, 2008). This comparative approach produces a more complete understanding of healthy cognitive function across human life span (de Kwaasteniet et al, 2013; Guye et al, 2010; Hahn et al, 2013; Salami et al, 2014; van den Heuvel and Fornito, 2014; Wang et al, 2016).

There is a complex relationship between brain structure and function (Rykhlevskaia et al, 2008; Suárez et al, 2020). Independent laboratories have found a striking similarity between patterns of white matter fibers and functionally meaningful parcellations of the cortex (Greicius et al, 2008; Johansen-Berg et al, 2004; Jung et al, 2017). For example, the most central nodes of functional networks are directly and strongly connected by white matter tracts (Greicius et al, 2008). Some studies have focused on a temporal association of activity across remote regions, which is interpreted as interaction across these regions and commonly referred to as functional connectivity (Friston, 2002). Studies that compare patterns of structural white matter connectivity and functional connectivity have found moderate correspondence in structural and functional connectivity (Honey et al, 2009; Parker et al, 2003; Sporns et al, 2011; Wang et al, 2013). This indicates that there are many regions that are not directly connected, but still can show functional interactions (Ashourvan et al, 2019; Hagmann et al, 2008; Honey et al, 2010; Honey et al, 2009; Liao et al, 2015; Røge et al, 2017; Sun et al, 2017; Thomas et al, 2009). This implies that there are regions that are indirectly connected with each other, and evidence demonstrates that accounting for indirect connections improves correspondence between structural and functional connectivity (Honey et al, 2009). This evidence illustrates that there is a complex and nontrivial relationship between brain structure and function. As a result of this complexity, it becomes challenging to interpret patterns of results in cognitive neuroimaging investigations when neural structure and function diverge, yet it is possible that divergence provides important information about the mechanisms involved.

Researchers have demonstrated that both regional and inter-regional relationships between brain structure and function can profoundly influence cognition in healthy and clinical populations. For example, one study investigated structural and functional differences across two aging groups with good and poor episodic memory (Persson et al, 2006). It was found that severe decline in episodic memory was uniquely associated with reduced integrity of white matter in the anterior part of the corpus callosum and increased activity in right prefrontal cortex during episodic encoding. It was argued that the unique activity in right frontal regions observed for the older group with memory impairment may have been a compensatory mechanism for the structural disruption. In another example, both structural connectivity (SC) and functional connectivity (FC) have both been found to be decreased in temporal lobe epilepsy patients compared to controls (Liao et al, 2011; Zhang et al, 2011). Furthermore, similarity between SC and FC has been found to be decreased in people with epilepsy (Chiang et al, 2015). In particular, this decoupling was then modulated by duration of epilepsy and structural changes to individual regions, which were unique to patients with left versus right temporal lobe epilepsy. Unique patterns of disruption of coupling between brain structure and function have been reported in other aspects of aging, including emotion processing, executive function, language, motor function inhibition (Ford and Kensinger, 2014; Hu et al, 2013; Mander et al, 2017; Ritchie et al, 2018; Sun et al, 2017), and clinical disorders, including schizophrenia, depression autism, stroke, dementia, and many others (Anderson et al, 2011; Carter et al, 2009; Cocchi et al, 2014; Hojjati et al, 2018; Wang et al, 2016; Weinstein et al, 2011). In addition, several regression studies suggest that the relationship between structure and function contributes unique variance to explanation of cognitive performance (Dhamala et al, 2021; Mansour et al, 2021; Rasero et al, 2021).

To provide an overview of trends and developments within this research field, the present systematic review assesses how researchers have attempted to combine structural and functional brain imaging data in healthy adult populations. The review considers the findings to date, relevant methodological considerations, and outstanding areas that need to be addressed. Through this we hope to gain a better understanding of the state of the field and highlight the potential of combining structural and functional neuroimaging data.

## Methodology

The present work has been conducted in accordance with the guidelines for systematic reviews (Moher et al, 2009). First, four research questions were formulated: (i) how many articles have included neuroimaging data and analysis of both structure and function from healthy adults, (ii) what proportion of articles identified by the first research question have also obtained and analyzed cognition, (iii) what proportion of articles identified by the second research question have quantitatively characterized the relationship between neural structure and function, and (iv) what methods of statistical analysis have been used to make the quantitative comparison between neural structure and function.

To answer these research questions, Web of Science and Scopus databases were searched on the 21st of October in 2021. The following terms were used to search across topics, titles, abstracts, and keywords: human brain, neuroimaging, structural, and functional. The following terms were explicitly excluded from the search as they imply clinical research: pathology, disease, syndrome, disorder, reviews. The following search string has been used in Web of Science: “((TS = (human brain AND neuroimaging AND structural AND functional)) NOT TS = (pathology OR disease OR syndrome OR disorder)) NOT TS = (review).” The following search string has been used in Scopus: [(TITLE-ABS-KEY (human AND brain AND neuroimaging AND functional AND structural) AND NOT TITLE-ABS-KEY (pathology OR disease OR syndrome OR disorder) AND NOT TITLE-ABS-KEY (review))]. Only formally published, peer-reviewed literature was included, and “gray literature” was excluded. In-text inclusion criteria were set to produce a report that is most representative of cognitive neuroimaging research conducted on healthy adult population. The full list of selection criteria, including article form, data analysis, study design, and populations, can be found in Supplementary Appendix A1. Articles were selected for this review with the following process: articles were identified from databases, duplicate articles were removed, titles and abstracts were screened, and finally in-text elimination was conducted. As part of in-text elimination process, the articles without cognitive outcome were excluded. Article selection process was conducted by Marta Czime Litwińczuk.

The following information was recorded during data collection: cognitive task, neuroimaging acquisition protocols and paradigms, neuroimaging data preprocessing, outcome measures of neuroimaging data, scales of neuroimaging analysis, and methods of integrating information about brain structure and function. These data are provided with Supplementary Data S1.

## Results

### Literature search and study characteristics

**FIG. 1.**
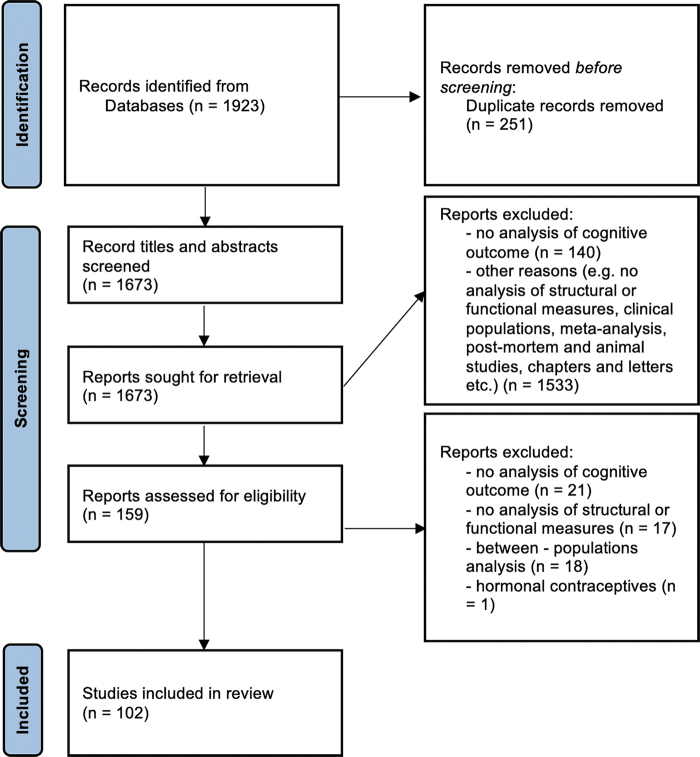
Flow diagram obtained from PRISMA guidelines for systematic reviews (Moher et al, 2009). It illustrates the literature search and literature selection process used in present review. PRISMA, Preferred Reporting Items for Systematic Reviews and Meta-Analyses.

The process of identification, screening, and selection of studies presented in Figure 1 has been obtained from Preferred Reporting Items for Systematic Reviews and Meta-Analyses (PRISMA) guidelines (Moher et al, 2009). First, 1,924 articles were identified during database search, and 251 records duplicates were removed. Then, 1,514 records were removed during screening of titles and abstracts. At this stage, 140 studies were removed as they relate structural and functional neuroimaging data without consideration of cognition, and 1,374 studies were removed for other reasons (e.g., studies lacking either structural or functional neuroimaging data, developmental, clinical, pharmacological, neurostimulation, animal, and postmortem studies, meta-analysis, chapters).

**FIG. 2.**
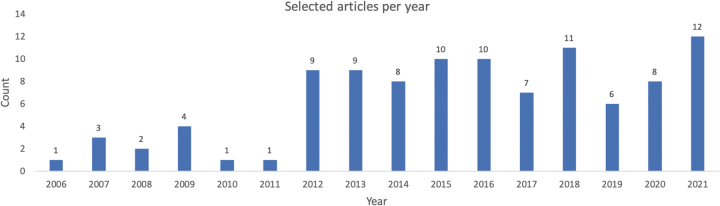
A time line represents count of selected publications for each year.

Finally, 159 articles were submitted to full-text assessment of eligibility. At this stage, 21 studies were removed as they relate structural and functional neuroimaging data without consideration of cognition. Overall, the selection process resulted with 102 articles that were included in our literature review. The articles that present structural, functional, and cognitive data in healthy young adult population accounted for 5% of initial search results. Figure 2 illustrates the number of identified articles for each year.

### Research avenues

The selected articles covered a wide variety of cognitive domains and their respective processes with many articles investigating multiple processes across many domains. Table 1 summarizes the frequency of cognitive processes across the selected literature. Overall, the most investigated cognitive domain was language (featured in 21 articles), followed by memory (featured in 20 articles) and working memory (featured in 19 articles).

**Table 1.** A Summary of Cognitive Processes Across Selected Literature

Domain	Count	Domain	Count
ACTION	1	MIND WANTERING	1
ARITHMETIC	3	MOTOR FUNCTION	8
ATTACHMENT	1	PERCEPTION	14
ATTENTION	7	PERSONALITY	4
CREATIVITY	1	PROCESSING SPEED	4
DECISION MAKING	8	REASONING	10
EMOTION	9	RISK	4
EXECUTIVE FUNCTION	11	SELF-AWARENESS	1
FACE PROCESSING	7	SLEEP	2
HYPNOSIS	2	SOCIAL COGNITION	10
INHIBITION	8	SPATIAL ORIENTATION	1
INTELLIGENCE	5	TIME PROCESSING	1
LANGUAGE	21	VISUAL	5
LEARNING	6	VISUOSPATIAL	6
MEMORY	20	WELLBEING	7
META-COGNITION	3	WORKING MEMORY	19

Occurrence of cognitive processes was counted, and if an article has investigated multiple cognitive processes then the count was the fraction of all processes featured in the article.

Table 2 summarizes the most popular research avenues for research to neural substrates of cognition. Mapping of structural and functional correlates of cognition was the most popular research avenue. Investigations into effects of individual differences were another popular research avenue. In this study, research explored the effects of biological factors, cognitive strategies, and demographics on the mapping of structure-cognition associations and function-cognition associations. Biological factors of interest included effects of genotype (Buckholtz et al, 2007; Chen et al, 2018; Filippini et al, 2009; Harneit et al, 2019), microbial gut profile (Tillisch et al, 2017), and hormonal cycles and changes (Lisofsky et al, 2015; Mujica-Parodi et al, 2014). Studies of confounding effects of demographic characteristics studied the effect of age, gender, handedness, height, and weight (Chialvo et al, 2013; Cuzzocreo et al, 2009; Han et al, 2020; Jiang et al, 2019; Lin et al, 2013; Sanfratello et al, 2014). Last category of individual differences included investigations of effect of unique cognitive strategies (Chialvo et al, 2013; Forstmann et al, 2008; Lin et al, 2013; Sanfratello et al, 2014). Some studies have assessed the ability to predict cognitive data from structural and functional information (Jiang et al, 2019; Rasero et al, 2021), and some studies have investigated the effect of structural and functional priors on analysis of cognition (Chica et al, 2017; Kohno et al, 2017; Xue et al, 2015). Both these research avenues explore how much complementary information about variation in cognitive performance is obtained from structural and functional information. Finally, some studies have compared the relationship between brain structure and function and cognition across different age groups (Bangen et al, 2012; Gur et al, 2010; Salami et al, 2014; Varol et al, 2018; Yoshimura et al, 2020), and some studies have assessed effects of training (Chialvo et al, 2013; Cuzzocreo et al, 2009; Han et al, 2020; Harneit et al, 2019; Jiang et al, 2019; Lin et al, 2013; Lisofsky et al, 2015; Sanfratello et al, 2014; Tillisch et al, 2017). These investigations mapped how age and training impact associations between brain structure, function, and cognitive performance.

**Table 2.** A Summary of Research Avenues Across Selected Literature

Research avenue	Count
Individual differences	15
Biological	9
Cognitive	4
Demographic	2
Individual differences, mapping of mechanism, prediction of cognitive performance	1
Biological	1
Individual differences, prediction of cognitive performance	2
Demographic	2
Life span changes	7
Mapping of mechanism	54
Mapping of mechanism, prediction of cognitive performance	1
Mapping of mechanism, prediction of cognitive performance, effect of priors on model statistics	1
Mapping of mechanism, effect of priors on model statistics	1
Plasticity	7
Plasticity, life span changes	1
Prediction of cognitive performance	5
Prediction of cognitive performance, effect of priors on model statistics	1
Effect of priors on model statistics	6

### Neuroimaging data and data analysis

The selected articles have shown an even balance between analysis of magnetic resonance imaging (MRI) and diffusion tensor imaging (DTI), but functional MRI (fMRI) dominated the research field (Table 3). The functional imaging research was dominated by task paradigms, but resting state paradigm has also featured in many articles and some articles combined task and resting state paradigms.

**Table 3.** A Summary of Structural and Functional Methods and Functional Paradigms

Modality category	Count	Modality category	Count
**DTI**	**45**	**T1 MRI**	**40**
**EEG**	**1**	**EEG**	**1**
Task	1	Task	1
**fMRI**	**41**	**EEG, fMRI**	**1**
Resting state	9	Task and resting state	1
Task	27	**fMRI**	**37**
Task and resting state	5	Resting state	16
**fMRI, GABA spectroscopy**	**1**	Task	18
Task	1	Task and resting state	3
**MEG**	**1**	**fMRI, MEGA-PRESS**	**1**
Task	1	Resting state	1
**PET**	**1**	**T1 MRI, DTI**	**11**
Resting state	1	**fMRI**	**11**
**DTI, magnetic resonance elastography**	**1**	Resting state	4
**fMRI**	**1**	Task	6
Task	1	Task and resting state	1
**DTI, quantitative MRI**	**1**	**T1, T2 FLAIR MRI**	**1**
**fMRI**	**1**	**fMRI**	**1**
Task	1	Resting state	1
**T1/T2 MRI**	**1**	**T2 MRI**	**1**
**fMRI**	**1**	**fMRI**	**1**
Task	1	Task	1
**T1/T2 MRI, DTI**	**1**		
**fMRI**	**1**		
Task and resting state	1		

Boldfont has been used to indicate neuroimaging modalities. Standard font has been used to indicate paradigm employed during functional imaging.

DTI, diffusion tensor imaging; EEG, electroencephalogram; FLAIR, fluid attenuated inversion recovery; fMRI, functional magnetic resonance imaging; GABA, gamma-aminobutyric acid; MEG, magnetoencephalography; MEGA-PRESS, mescher–garwood point resolved spectroscopy; PET, positron emission tomography.

### Methods of integrating structural and functional data

Studies were categorized by the types of inference they made about the relationship between brain structure and function: (i) indirect, (ii) semidirect, and (iii) direct. Table 4 summarizes prominence of each inference type and further detail regarding semidirect and indirect inferences, and the following paragraphs define each type of inference and provide examples of conceptual questions that they have been applied to.

**Table 4.** A Summary of Different Types of Inference Made to Understand Relationship Between Brain Structure and Function, and Cognition

Inference type	Count
**Indirect inference**	**51**
**Semidirect inference**	**16**
Function informed ROI analysis	7
Shared measure	3
Structure informed ROI analysis	6
**Direct inference**	**31**
Effect of structural priors on model	1
Effect of structural priors on model, fusion	1
Inferential statistics of distance	1
Joint model	7
Joint model, fusion	2
Mediation	1
Predictive model	1
Ratio	1
Similarity	14
Similarity, joint model	1
Similarity, overlap	1
**Direct and semidirect inference**	**4**
Effect of priors and function informed ROI analysis	1
Ratio and structure informed ROI analysis	1
Similarity and function informed ROI analysis	2

Boldfont has been used to indicate inference type. Standard font has been used to provide detail on analysis used to support the inference.

ROI, region of interest.

First, indirect inference referred to studies that conducted separate analysis of brain structure and function without any quantitative evidence of their relationship. It was the most common type of inference, featured in 51 out of 102 selected studies. Most commonly, indirect inferences were made to assess if structural-cognitive associations show a spatial overlap with functional-cognitive associations. This process allows authors to infer whether there is a functional relevance to any structure-cognition associations. Research in this area has effectively managed to demonstrate spatially shared and unique structural and functional substrates of cognition (Porcu et al, 2021; Sala-Llonch et al, 2015; Tavakol et al, 2021; Wiech et al, 2014). In addition, studies using indirect inference have demonstrated that training-related and aging-related changes in brain structure and function both relate to changes in cognitive abilities (Gryga et al, 2012; Yang et al, 2019). Finally, studies have demonstrated unique structural and functional associations with effects of biological factors (e.g., genetic, hormonal balances, microbial gut profile), demographics (e.g., handedness, gender, age), and cognitive strategies (Chialvo et al, 2013; Cuzzocreo et al, 2009; Han et al, 2020; Harneit et al, 2019; Jiang et al, 2019; Lin et al, 2013; Lisofsky et al, 2015; Sanfratello et al, 2014; Tillisch et al, 2017). Overall, studies using indirect inference have focused on exploring spatially shared and unique structural and functional substrates of cognition. However, this was done without exploring the extent to which structure and function relate to each other or the importance of shared substrates of cognition.

Second, semidirect inference referred to studies where authors have not provided statistical analyses of how brain structure and function relate to each other, but the analyses of each modality allowed some inference about how the modalities can relate. It was the least common type of inference, featured in 16 out of 102 selected studies. Studies in this category have obtained common measures of properties of both structure and function without formally testing how these measures relate across modalities. For example, rich club coefficient was obtained and has not been formally related, but it demonstrated that central cortical hubs are shared across structure and function and these hubs facilitate outgoing effective connectivity (Senden et al, 2018). Thus, use of shared measures of cortical organization has been implemented to identify shared roles of structural and functional networks. In another example of semidirect inferences, authors have used the topological location of results obtained for one modality to narrow down analysis of the other modality through informing the location of regions of interest (ROIs) (Adnan et al, 2015; Beer et al, 2013). Table 4 illustrates prominence of function-driven and structure-driven definition of ROIs. Similarly, to indirect investigations, these investigations reveal a functional relevance of structural correlates of cognition and vice versa. These investigations may miss on revealing unique substrates of cognition outside of data-driven ROIs. However, implementation of this analysis has largely aimed to explore structural connectivity patterns underlying functionally active regions to assess whether specific patterns of structural connections underlie task-specific activation (Grotheer et al, 2019; Hoeren et al, 2013).

Third, direct inference was defined as inference made with quantitative evidence to support the interpretation of the relationship between structural and functional correlates of cognition. It featured in 31 out of 102 selected studies. The variety of direct inferences in quantitative analyses across studies is shown in Table 4. Analysis of similarity (e.g., correlation, cosine similarity) was the most common method of directly comparing brain structure and function. This adds to the mapping of neural substrates of cognition with direct, quantitative evidence whether overlapping patterns of structural and functional substrates of cognition are related to each other (Chavan et al, 2015; Han et al, 2020; Jung et al, 2018; Wang et al, 2021; Zhang et al, 2016). Next, joint models were constructed by studies that used structural and functional data to predict cognitive outcomes (Bajaj et al, 2021; Jiang et al, 2019; Rasero et al, 2021). Joint models have been implemented to assess whether joint consideration of structural and functional information reveals additional information about the variation in cognitive performance. Further exploring the idea that multimodal information may improve modeling of cognition, two studies have assessed the effect of structural priors on functional model evidence (Kohno et al, 2017; Xue et al, 2015). Following the same question with another method, one study attempted to predict functional network characteristics using structural information, to assess how much variation in cognition and functional network activity can be explained by structural information (Chica et al, 2017). These approaches can effectively explore the question whether structural information complements and further improves quality of functional models of cognition. Another means to assess how much functional substrates of cognition may rely on structure has been estimation of what ratio of functional connections involved with cognition had underlying direct structural connections (Sokolov et al, 2018a; Sun et al, 2012). Furthermore, Noonan et al (2018) have not only assessed how much overall functional network relies on structural brain features but also the authors have assessed whether specific functional networks are more closely related to brain structure than others. The authors have statistically assessed the extent of overlap between clusters of neural substrates of cognition for each modality. Thus, they could differentiate the relationship between structure and specific functional networks. One study has used inferential statistics to assess the difference in organization of structural and functional networks, to formally explore how organization of structural and functional networks differs (Jung et al, 2018). This approach allowed authors to explore what structural and functional features relate to cognition to develop an understanding of organizational features that may support cognitive performance. Finally, to identify joint directions of variance between structure and function, some research groups have conducted data fusion such as independent component analysis (Bolton and van De Ville, 2020; Lerman-Sinkoff et al, 2017). This allows authors to relate structure and function to each other before relating them together to cognition. Such approach is also similar to correlation analysis in that it has been implemented as a mapping tool to identify shared and unique features of structure and function, but it reduces the multiple comparison problem and need for adjustments of *p*-values.

## Discussion

The relationship between brain structure and function appears to have profound consequences for understanding cognition (Ford and Kensinger, 2014; Hu et al, 2013; Jandric et al, 2021; Mander et al, 2017; Ritchie et al, 2018; Sun et al, 2017). In this systematic review we determined how structural and functional neuroimaging methods have been integrated to study cognitive function and adaptive behavior. A search was conducted across two databases in accordance with PRISMA guidelines (Moher et al, 2009). We assessed the prevalence of studies combining structural and functional neuroimaging data for explaining cognition and evaluated their choice of methods. The results demonstrate that there are to date relatively few studies attempting to combine structural and functional neuroimaging data, and most studies that use that do use indirect methods to infer the relationship between brain structure and function without formally relating these measures. In this study, we consider what these findings mean for the field and how the shift toward direct inference with quantitative methods can lead to greater insight into how the structure and function of the brain combine to effect cognition.

First, this systematic review assessed the prevalence of studies that present structural, functional, and cognitive data in healthy young adult population. Only 5% of the initial search results (102 out of 1,924 studies) have examined links between structural and functional data and cognition in healthy adults, and 161 neuroscientific studies were removed as they related structural and functional neuroimaging data without consideration of cognition. Investigations that address this explore how structure and function that serve cognitive function have the potential to produce more complete understandings of healthy cognitive function than unimodal analysis. Combining brain structure and function information explains more variance in cognitive performance than either modality alone (Dhamala et al, 2021; Jiang et al, 2019; Rasero et al, 2021). These studies can also provide new insight, by determining, for example, causal interactions between regions (Sokolov et al, 2018a; Sokolov et al, 2018b) or how new learning and training can result in neuroplasticity (Sun et al, 2016; Yang et al, 2019).

Understanding relationships between brain structure, function, and cognition can also provide insight into how these relationships breakdown in neurological and psychiatric disorders. The present review's selection criteria eliminated investigations of atypical populations, but some of the selected articles were then implemented in investigations into mechanisms of disease and recovery. To illustrate, Yang et al (2019) investigated the effects of mindfulness training and were considered in later studies of general well-being of healthy populations (Tortella et al, 2021), improvements in cognitive function of diabetic patients (Alipor et al, 2019), and recovery from depression (van der Velden et al, 2022). In another example, Jung et al (2018) investigated how organization of structural and functional connectivity relates to language, work that had direct implications in understanding language deficits in semantic variant primary progressive aphasia (Battistella et al, 2019) and temporal lobe epilepsy (Black, 2020). This illustrates the potential impact from a deeper understanding of how brain structure and function integrate.

Having explored the prevalence of research on neural substrates of cognition in the literature, the present review considered methods for integrating across modalities. Three approaches to relating structure and function were observed: (i) a direct inference based on quantitative evidence, (ii) semidirect inference based on closely related or similar processing steps, (iii) indirect inference based on separate analysis of the two approaches. Indirect inference was the most common approach. During indirect inference, experimenters initially investigated how brain structure or function impacted cognitive function, and next they inferred the degree of similarity between the two modalities. While indirect inference is a simple approach, it is very powerful in addressing a wide variety of research questions. Most commonly, indirect inferences were made to assess if structural-cognitive associations show a spatial overlap with functional-cognitive associations. Research in this area mapped spatially shared and unique structural and functional substrates of cognition (Porcu et al, 2021; Sala-Llonch et al, 2015; Tavakol et al, 2021; Wiech et al, 2014). Furthermore, some studies using indirect inference have also demonstrated that cognitive training and aging both show overlapping changes in brain structure and function that both relate to changes in cognitive abilities (Gryga et al, 2012; Yang et al, 2019). Interestingly, studies comparing structural and functional associations with cognition have shown more overlap in healthy elderly adults in the absence of effects in young adults (Bangen et al, 2012; Gur et al, 2021; Salami et al, 2014; Varol et al, 2018; Yoshimura et al, 2020). Thus, indirect inference has been effectively implemented to demonstrate that neural changes across life span result with increased coupling between structure-cognition and function-cognition associations. Additional lines of related research that used indirect inferences include investigations of individual differences. Research in this area highlights that confounding effects of biological variables, demographics, and cognitive strategies have different effects on structural and functional associations with cognition (Chialvo et al, 2013; Cuzzocreo et al, 2009; Han et al, 2020; Harneit et al, 2019; Jiang et al, 2019; Lin et al, 2013; Lisofsky et al, 2015; Sanfratello et al, 2014; Tillisch et al, 2017). However, while many conceptual advancements have been made using indirect inferences, studies using indirect inference have not quantified how strong the relationship between structure and function is.

Similarly, semidirect inferences were implemented to further explore if regions that are functionally related to cognition share structural connections (Adnan et al, 2015; Beer et al, 2013). This has demonstrated that regions active during task tend to share white matter connections but the identified fibers may also extend to other areas that are not currently involved with a task. Neuroscientific evidence demonstrates that functional networks can change their configurations in response to specific task demands (Cohen and D'Esposito, 2016; Cole et al, 2014; Cole et al, 2013; Krienen et al, 2014; Salehi et al, 2020). Thus, semidirect inferences have effectively complemented neuroscientific research with evidence that task-specific network organization relies on structural scaffolding of white matter connections. However, again these studies are unable to make inference about how important is this structural scaffolding for cognition. This means that research has focused on exploring the similarities and differences between structural and functional correlates of cognition, but it is still largely unexplored how much the nature of this relationship impacts cognitive performance.

The present review also focused on methods used to make direct inferences. We can roughly place these methods under the umbrellas of four approaches; (i) comparative approach, (ii) predictive approach, (iii) fusion approach, and (iv) complementary approach. First, the comparative approach assesses differences in characteristics of each network through measures of distance and significance testing. We have found that data were most commonly related across modalities with analysis of similarity such as correlation and quantification of results cluster overlap (Noonan et al, 2018). They were used to assess both linear and nonlinear relationships between a variety of structural and functional properties. Structural properties included volumetric values like cortical thickness and cortical surface area (Rasero et al, 2021), microstructure like gray and white matter directionality (e.g., fractional anisotropy) (Chavan et al, 2015), white matter connectivity like number of tract streamlines connecting region pairs (Sokolov et al, 2018b), and symmetry scores assigned to reflect how symmetric these properties are across the two brain hemispheres (Josse et al, 2009). Functional properties have included properties of evoked activity like strength, count of activated voxels and laterality of activation (Zuo et al, 2016), and strength of FC as reflected by the correlation in signal intensities across remote regions (Rasero et al, 2021). Finally, inferential statistics have been used to assess if organizational characteristics of structural and functional networks significantly differ (Jung et al, 2018).

The second approach of relating brain structure and function involves production of predictive models of cognition. Authors have used multiple regression (Ford and Kensinger, 2014; Jung and Kim, 2020; Putnam et al, 2008), canonical correlation analysis (Han et al, 2020; Lerman-Sinkoff et al, 2017), partial least squares (Dzafic et al, 2019), and connectome-based predictive modeling (Jiang et al, 2019). One study started with sparsity-constrained principal component regression in each modality, and the selected features of each modality were fed-forwards to a lasso analysis that integrated the models to a single model (Rasero et al, 2021). Another study produced predictions of cognitive performance separately using structural and functional connectivity and then calculated the average of the two predictions (Bajaj et al, 2021).

Third, the fusion approach was observed where structural and functional information was fused before relating them to cognition. One such approach involved independent component analysis, which was conducted on both connectivity sets and then canonical correlation analysis conducted on the resulting components (Lerman-Sinkoff et al, 2017). In another study, brain structure and function were fused, where structural connectivity produced a prior distribution of functional connectivity, which was then related to cognition (Xue et al, 2015).

Finally, the complementary approach used information about structural characteristics to better understand the functional models of cognition. For example, one study evaluated how much additional variance is explained when structural priors are added to functional models (Kohno et al, 2017). Another group assessed if structural characteristics can be predictive of functional neural interactions observed between cognitive tasks (Chica et al, 2017). Finally, Sun et al (2012) calculated the ratio of the effective connectivity observed during tasks with underlying direct structural white matter connections. It is important to note that some work may use several of these four general approaches. For example, partial least squares method can be used to fuse structural, functional, and cognitive data, because it finds linear combination of predictor variables that covary with the response variable and projects all the information into a new space. Thus, we see that this method can both be used as a predictive and as fusion method depending on the kinds of research questions that the authors wish to address.

Each one of these approaches has their strengths in addressing specific research questions. The comparative approach allows us to understand what properties differ across structure and function. Consequently, the interpretation of results is easier and more meaningful as it explains what makes each modality unique and why unique patterns of results may be observed. However, it is important to remember that specific features of functional connectivity may be more difficult to be compared against features of brain structure. For example, there is evidence to demonstrate that there is indirect functional connectivity, where functional connections can be observed in the absence of direct structural connections. In addition, functional connectivity can be negative, where a pair of remote regions shows a negative association in their signal strength. It is currently unclear how these unique functional characteristics should be compared against structural characteristics. Investigations that undertake complementary approach may be more suited to explore how much of brain function is related to brain structure, as they explore how prior information about brain structure can impact on the relationship between brain function and cognitive performance. The limitation of such investigations is however that they reflect little information about how neural function shapes neural structure, thus they provide a one-sided view of the relationship. Next, the predictive and fusion approaches have the capacity to effectively approach the multivariate nature of this research field. For example, Rasero et al (2021) have started from a series of univariate regression models, composed of either structural or functional information, and implemented a stacked approach to eventually develop a combined model that is multivariate. In another example, Dhamala et al (2021) have produced three regression models; using only structural connectivity, using only functional connectivity, and using both. The challenge of such approaches remains that they may witness suppressor variables, where the variance in the response data accounted for by one variable may impact the beta weights of another variable (Lancaster, 1999). In addition, to our knowledge, so far research has focused on construction of linear models, while interactions between structure and function have remained unexplored. Research into mediation effects in regression models will be necessary to more fully explore how the relationship between brain structure and function serves cognitive function. Thus, we see that every approach can be used to answer slightly different questions about the relationship between brain structure and function, and authors can be creative in how they integrate several approaches to produce very refined models of cognition.

Implementation of direct inferences has further elucidated how the relationship between brain structure and function impacts cognitive performance. Measures of similarity that include correlation coefficients, extents of overlap, and inferential statistics have demonstrated that distinct organizational properties of structural and functional networks may explain divergent substrates of cognition (Jung et al, 2018; Wang et al, 2021; Zhang et al, 2011). Furthermore, latent components that reflect related multimodal features of the brain correlate with cognitive performance and demonstrated related neural substrates of specific cognitive domains (Lerman-Sinkoff et al, 2017; Xue et al, 2015). However, direct inference is not limited to mapping of overlapping substrates of cognition. To illustrate, direct comparative analysis has revealed that aging induces not only spatially overlapping but also correlated structural and functional changes that are also associated with sensorimotor skills (Fling et al, 2012). In another example, demographic information has demonstrated an association with cortical thinning and functional activation, and these factors carried impact on positive behavioral traits (e.g., fluid intelligence and life satisfaction) (Han et al, 2020). Furthermore, Chavan et al (2015) have highlighted that inhibitory control training elicits functional activation changes but these effects may not be substantial enough across all sites to elicit associated structural changes. Thus, direct approach has demonstrated that overlap between neural substrates of cognition do not necessarily imply correlation between structural and functional features. Furthermore, it appears that structural and functional features capture complementary information about the state of the system which may improve our understanding of healthy cognitive function. For example, predictive models have demonstrated that for some but not all cognitive domains the multimodal predictions of cognitive performance are greater than the sum of their parts (Jiang et al, 2019; Rasero et al, 2021). In other examples, introduction of structural priors to functional modeling of cognition improved efficiency of functional models (Chica et al, 2017; Kohno et al, 2017; Xue et al, 2015).

This review also considered what data were acquired and how it was prepared for analysis. This highlighted a number of limitations. First, it became apparent that fMRI protocols have taken clear dominance over other functional imaging techniques in this research field. As mentioned in the introduction of this review, fMRI method suffers from low temporal resolution and is not a direct measure of neural activity. It is essential to dedicate more research in the future to neuroimaging data with higher temporal resolution, such as EEG. This would allow more direct study of neural signals with millisecond precision. Consequently, signals that are not effectively reflected in the BOLD response could be studied, such as mismatch negativity which occurs 150 msec following stimulus onset and is recognized as a marker of detection of stimulus irregularity (Näätänen, 1995). Second, there was a clear dominance of experimental protocols using cognitive task performance over resting state fMRI. Resting state has been subjected to scrutiny and debate, as mental state and mental processes of the subjects are uncontrolled (Damoiseaux et al, 2006; Poldrack and Devlin, 2007; Smith et al, 2009). In contrast, task paradigms are carefully designed to engage and manipulate a cognitive process of interest and it is more clear what mental state was evoked in participants. However, resting state paradigms show moderate to high test-retest reliability and replicability across datasets and laboratories (Biswal et al, 2010; Buckner et al, 2009; Shehzad et al, 2009; Zuo et al, 2010a; Zuo et al, 2010b). This means that resting state may allow easier comparison of results across independent research laboratories. Furthermore, resting state produces consistent activation of a specific set of regions known as default mode network (Greicius et al, 2002). The function of this network has been related to cognitive functions, including but not limited to task switching, learning, and social cognition (McCormick and Telzer, 2018; Smith et al, 2018; Spunt et al, 2015). Furthermore, its abnormal function has been implicated in many disorders such as dementia, schizophrenia, epilepsy, anxiety and depression, and autism (Broyd et al, 2009). Third, many studies have used correlation analysis as a method of relating brain structure and function. However, mediation analysis and partial correlation analyses were largely not used. This is problematic, because it has been demonstrated that two regions can display functional connectivity in the absence of direct structural links between them, and the similarity between functional and structural networks increases when indirect structural links are permitted in the analysis (Hagmann et al, 2008; Honey et al, 2009). This means that studies which ignore indirect links between regions may find less similarity between brain structure and function than studies that would account for those links.

To conclude, the present review was conducted to survey the prevalence of studies integrating brain structure and function for understanding cognition and detail the methods used in these analyses. Integrating structure and function and cognition is key for a full understanding of brain function and cognitive function through life span, disease, and recovery. This review demonstrated that the relationship between brain structure and function and cognitive function is still largely underexplored. Inferences about the relationship between neural structure and function and cognitive function were indirect, semidirect, or direct, depending on what kind of evidence was used to support the interpretation of that relationship. Direct inference was not as common as indirect inference, and we have provided a brief discussion of available and previously used approaches to handling this multivariate analysis.

## Data Availability

The articles selected during the systematic review process and data collected from these articles are available as part of Supplementary Data.

## Supplementary Material

Supplemental data

Supplemental data

## Supplementary Material

Supplementary Data S1

Supplementary Appendix A1
