# Supplementary material for: Relating Cognition to both Brain Structure and Function: A Systematic Review of Methods
Source: Brain Connect. 2023 Apr 4;13(3):120–32. doi: 10.1089/brain.2022.0036 (PMC10079251; doi:10.1089/brain.2022.0036)
Supplement: Supplemental data [file Supp_AppendixS1.docx]

### Supplementary Material 1A

### Selection Strategy

The goal of selection criteria was to produce a report that is most representative of cognitive neuroimaging research conducted on healthy adult population. Following sub-sections outline how elimination was conducted based on data form and data analysis, study design and populations presented in articles.

*Data form and data analysis*

We searched for articles from peer-reviewed journals that were accessible in English language and analysed primary data or data from sharing initiatives. The first elimination criteria were set to remove studies that lack cognitive outcome measures, structural neuroimaging data or functional neuroimaging data. Further, we eliminated studies that have collected both structural and functional data but have only conducted analysis of one of those neuroimaging methods.

Subsequently, we also removed any studies that obtained data from animal work, prenatal development, computational simulations or automated data synthesis platforms. Furthermore, we removed articles that were in the form of case-studies, meta-analyses, reviews, chapters, editorials, correspondence or letters.

However, we included methodological articles reporting, discussing and advocating novel methods of data analysis and algorithms, if those articles were found in-text to conduct analyses of primary data and to be motivated by properties of neural physiology.

*Study design*

Many studies have been designed to assess impact of perturbing the neural system. Exclusion criteria were set for studies that have perturbed the chemistry of neural system, i.e. studies that involved administration of magnetic or electrical stimulation to the head or nerves, administration of drugs and medication, as well as disturbance of neural chemistry through sleep deprivation. Conversely, we included in our selection those studies that perturbed the system through behavioural changes, such as conditioning, training and learning of new skills (e.g. maths training, motor learning).

*Populations*

Finally, elimination criteria were defined for atypical populations and studies of lifespan-changes. We eliminated studies presenting samples from atypical populations, even if authors had included data from a control sample from typical population, because such sample usually carefully matches the atypical population of interest and cannot be representative of wider population. The category of atypical populations included clinical populations, populations of recovery and pre-term born population. Further, we excluded populations with history of dependence and addiction to substances and activities. For example, smokers, drug users etc. We also decided to exclude any population that had incidental exposure to substances, such as pre-natal exposure. Finally, for atypical populations, we also decided to exclude studies of populations that have suffered psychological trauma from stressful life events (e.g. natural disaster, military service).

Our objective was to study adult population; therefore, elimination of studies of lifespan changes was considered. Due to extensive changes in brain structure, chemistry and function associated with brain development and ageing (e.g. cited in main manuscript Smith et al., 2019), we have decided to focus on healthy adult population in age range 18-65. Following this goal, we eliminated reports that only presented samples falling outside of our selected age range. However, if lifespan studies presented samples inside our defined age range, then we assessed whether within group analyses had been conducted on young and middle adults. Thus, we included those studies that provided such within group analyses.
